# Buc Maintains Maternal RNA Stability and Embryogenesis in Zebrafish

**DOI:** 10.3390/cells14231879

**Published:** 2025-11-27

**Authors:** Ran Miao, Yan Xie, Qingqing Han, Yinglu Meng, Qin Tang, Jie Mei, Fan Ren

**Affiliations:** 1 Hubei Hongshan Laboratory, College of Fisheries, Huazhong Agricultural University, Wuhan 430070, China; 2 Key Laboratory of Breeding Biotechnology and Sustainable Aquaculture (CAS), Hubei Hongshan Laboratory, The Innovative Academy of Seed Design, Institute of Hydrobiology, Chinese Academy of Sciences, Wuhan 430072, China

**Keywords:** Bucky ball, Igf2bp3, maternal mRNA, embryo development, zebrafish

## Abstract

The maternal-to-zygotic transition (MZT) is a fundamental process in vertebrate embryogenesis, involving the clearance of maternal mRNA and activation of the zygotic genome. Orchestration of maternal mRNA stability ensures early embryogenesis. Recently, some germ plasm (GP) factors have been demonstrated to regulate the stability of maternal mRNA. Bucky ball (Buc) functions as a zebrafish GP organizer. However, it remains unclear whether Buc also protects maternal mRNAs from widespread decay in early embryos. Here, we report that overexpression of *buc* results in delayed maternal mRNA degradation and a concomitant delay in embryonic development, whereas *buc* knockout leads to accelerated maternal mRNA degradation and severe developmental defects, suggesting that both gain and loss of *buc* perturb early developmental programs. Mechanistically, this regulatory mechanism of Buc on maternal mRNA is mediated through the expression of RNA-binding protein Igf2bp3. Together, our findings suggest that the GP organizer Buc may stabilize maternal mRNAs in coordination with Igf2bp3, thereby contributing to the maintenance of maternal mRNA required for proper embryonic development during the MZT. This study expands the functional scope of Buc beyond GP assembly and reveals its critical role in safeguarding maternal mRNA integrity to ensure proper embryo development.

## 1. Introduction

Embryogenesis begins with a single totipotent cell that ultimately produces many specialized cell types that differ in function, morphology, and spatial arrangement. This process is primarily driven by dynamic changes in gene expression [1]. The first major transition in gene expression is the MZT, in which maternal mRNAs are cleared and zygotic gene activation occurs in vertebrate embryos [2,3,4,5,6,7]. Although most cell fates are permitted to emerge through zygotic genome activation during embryonic development [8], the differentiation of primordial germ cells (PGCs) depends on the stable retention of maternal mRNA in species that utilize preformed GP [9,10,11]. Across different organisms, a range of GP regulators have been identified as regulators of mRNA stability. In Drosophila, the GP component Pgc maintains mRNA stability by repressing miRNA activity [12,13]. Furthermore, a genome-wide analysis suggests that the RNA-binding protein Smaug or AU-rich element-binding proteins control maternal mRNA degradation, respectively [10]. In zebrafish, the RNA-binding protein Dead end 1 (Dnd1) counteracts *miR-430* function by binding to and protecting mRNAs from *miR-430*-mediated repression [14,15]. Furthermore, the RNA-binding protein Nanos binds the transcript of CNOT6, thereby protecting a specific subset of maternal mRNAs from degradation in echinoderms [16,17,18].

One RNA-binding protein, Igf2bp3, promotes its target mRNA stability. In zebrafish, Igf2bp3 binds and maintains a subset of maternal mRNA in early embryos. Disruption of maternal *igf2bp3* accelerates this mRNA degradation, resulting in cytoskeletal organization disruption, cell division impairment, and developmental arrest [19,20].

Moreover, Igf2bp3, together with Buc, is involved in GP assembly by regulating the stability of maternal GP RNAs [21]. Since Igf2bp3 regulates early embryo development and GP assembly by maternal RNA or maternal GP-RNA stability, maternal RNA (including maternal GP-RNA) is thought to be essential for these two events. However, regarding Buc functions in embryo development and GP assembly in early embryogenesis, whether this germ plasm organizer regulates maternal RNA stability remains elusive.

Here, we provide evidence that Buc promotes maternal mRNA stability via an Igf2bp3-expression-dependent pathway in early embryogenesis. Overexpression of *buc* delayed maternal mRNA decay and embryonic development by upregulating the expression of Igf2bp3. Conversely, loss of *buc* function led to accelerated mRNA decay and defective embryonic development in M*buc* (maternal-effect *buc* mutant), due to a downregulation of Igf2bp3 expression. Through gain- and loss-of-function analyses, we investigated how Buc works with Igf2bp3 to regulate maternal mRNA stability and safeguard essential maternal mRNAs for early embryonic development. This work expands the known functional repertoire of Buc as a GP regulator and furthers our understanding of the molecular mechanisms governing maternal mRNA stability in zebrafish embryos.

## 2. Materials and Methods

### 2.1. Zebrafish Maintenance

AB line wild-type (WT) zebrafish were maintained at 28.5 °C on a 14 h light/10 h dark cycle. Embryos were staged and collected according to standard procedures. All fish were maintained in accordance with the requirements of the Institutional Animal Care and Use Committee (IACUC) of Huazhong Agricultural University.

### 2.2. Generation of Mutant Lines

CRISPR/Cas9 was employed to produce *buc* mutants. The *buc*-specific target sequence was designed via an online platform (CRISPRscan: CRISPRs in vivo). PCR amplification with synthesized gRNA-F primers: GTAATACGACTCACTATAGGCAGGCGAAGGATTTGATAGTTTTAGAGCTAGAAATAGC and gRNA-R: AAAAGCACCGACTCGGTGCC produced the DNA templates for gRNA transcription. Following purification, the PCR products were used as templates for in vitro transcription with the Transcript Aid T7 High Yield Transcription Kit (Thermo Scientific, Waltham, MA, USA).

For Cas9 mRNA preparation, the Cas9 plasmid was digested with XbaI, followed by purification; the linear fragment was then transcribed using the T7 mMESSAGE mMACHINE kit (Ambion, Foster City, CA, USA). Both Cas9 mRNA and gRNA underwent lithium chloride precipitation for purification. Zebrafish embryos at the 1-cell stage were co-injected with 300 pg of Cas9 mRNA and 20 pg of gRNA. Genotyping was performed using the primer pair vF: GAGGTTGCCACTAAAGAA and vR: AGACACTCTGGCGCTCTT.

### 2.3. Overexpression Analysis

To produce mRNA, DNA fragments encoding full-length Buc fused with RFP at the C-terminus (*buc-rfp*) or RFP alone (*rfp*) were subcloned into the pCS2+ vector. Capped mRNAs were synthesized using the mMESSAGE mMACHINE SP6 kit (Ambion, USA). For overexpression experiments, approximately 1 nl of *buc-rfp* or *rfp* mRNA was microinjected into WT embryos at the 1-cell stage.

### 2.4. Western Blot

Embryos were treated with pronase E and buffer 1 (55 mM NaCl, 108 mM KCl, 1.25 mM NaHCO_3_) to remove both chorion and yolk. Between 50 and 100 embryos were then collected and lysed in 300 µL ice-cold lysis buffer composed of 50 um Tris (pH 7.5), 150 mM NaCl, 1 mM EDTA, 10% glycerol, 1% Triton X-100, and protease inhibitors (Sigma, St. Louis, MO, USA, P8340). Equal amounts of total protein were separated on 10% SDS-PAGE gels, transferred to PVDF membranes, and immunodetected using anti-Igf2bp3 and anti-β-actin (ABclonal, Wuhan, Hubei, China, AC026). HRP-conjugated anti-rabbit IgG (H+L) and ECL substrate (BIO-RAD, Hercules, CA, USA, 170-5061) were used for visualization. All embryos were collected strictly based on developmental time to ensure consistency across experiments.

### 2.5. Quantitative RT-PCR

Total RNA from embryonic samples was prepared with TRIzol reagent and DNase I treatment (Promega). To examine maternal gene expression during development, RNA was reverse-transcribed into cDNA using the PrimeScript RT Kit (Takara, Kusatsu, Shiga, Japan), which contains a mixture of Random 6-mer and Oligo(dT) primers. Quantitative RT-PCR was carried out using the Light Cycler^®^ 480 DNA SYBR Green I Master Mix (Roche, Basel, Switzerland) on the LightCycler^®^ 480 instrument. *β-actin* was employed for normalization. All experiments were performed in triplicate, and expression levels were calculated using the 2^−ΔΔCt^ method. Gene-specific primers are listed in Table 1. All embryos were collected strictly based on developmental time to ensure consistency across experiments.

### 2.6. Immunofluorescence

Embryos were fixed in 4% PFA for whole-mount immunofluorescence and processed with anti-Buc (1:100) and anti-Igf2bp3 (1:200) antibodies following established protocols. Confocal images were obtained with a Leica TCS SP8 microscope (Leica Microsystems, Wetzlar, Hesse, Germany) using the 3D view setting in LAS X software (Version 4.3) [20]. All embryos were collected strictly based on developmental time to ensure consistency across experiments.

### 2.7. RNA-seq and Data Processing of High-Throughput Sequencing

Total RNA was isolated from embryos at selected developmental stages using TRIzol reagent. Library preparation was performed with the KCTM Digital mRNA Library Prep Kit (Seqhealth Tech. Co., Ltd., Wuhan, China), using total RNA as the starting material and adhering to the manufacturer’s guidelines. This library system uses 12-base unique molecular identifiers to mark individual cDNA molecules, thereby minimizing amplification and sequencing biases. As part of the library preparation process, PCR-amplified fragments sized approximately 200–500 bp were enriched. The enriched libraries were quantified and sequenced on a DNBSEQ-T7 platform (MGI) using the PE150 sequencing mode to generate paired-end reads. All embryos were collected strictly based on developmental time to ensure consistency across experiments.

### 2.8. Protein Structure and Molecular Docking Analysis

Molecular docking analysis was performed using the AphaFold3 server (https://alphafoldserver.com/, accessed on 28 April 2025), and the docking data were visualized by PyMOL software (Version3.1.5.1).

### 2.9. Antibody Production

The sequence of the Buc-coding region at residues 1–242 was cloned into a pET32a expression vector. Then, the recombinant protein (His-tagged fusion protein) was expressed in *E. coli* and purified using Ni-NTA affinity chromatography with His-tagged. The purified Buc (1–242) recombinant fragment was used to immunize rabbits four times at intervals of 28, 14, and 14 days. Freund’s complete adjuvant (FCA) was used for the primary immunization and the rest of the injections. Five days after the fourth injection, antiserum titers were tested by ELISA. The rabbit exhibiting the highest titer was euthanized and bled on day 64 after the initial immunization.

For affinity purification, 1 mg of the purified Buc (1–242) fragment was coupled to CNBr-activated Sepharose 4B (GE) to generate the antigen affinity column. Immune serum was applied to the column, and antigen-specific IgG was eluted with glycine–HCl (pH 2.5), followed by immediate neutralization, dialysis, and concentration.

### 2.10. Statistics and Reproducibility

Independent biological replicates were used for all experiments, and replicate outcomes showed strong consistency. Information on sample numbers and grouping is listed in the figure legends and source data. Statistical analyses were performed with R and GraphPad Prism 7 using two-sided Wilcoxon tests, Mann–Whitney tests, or unpaired Student’s *t*-tests as appropriate. Error bars represent the standard deviation. Significance levels were set at *p* < 0.05 (*), *p* < 0.01 (**), and *p* < 0.001 (***), respectively.

## 3. Results

### 3.1. Overexpression of Buc Causes a Delay of Maternal mRNA Clearance and Embryo Development

**Figure 1:**
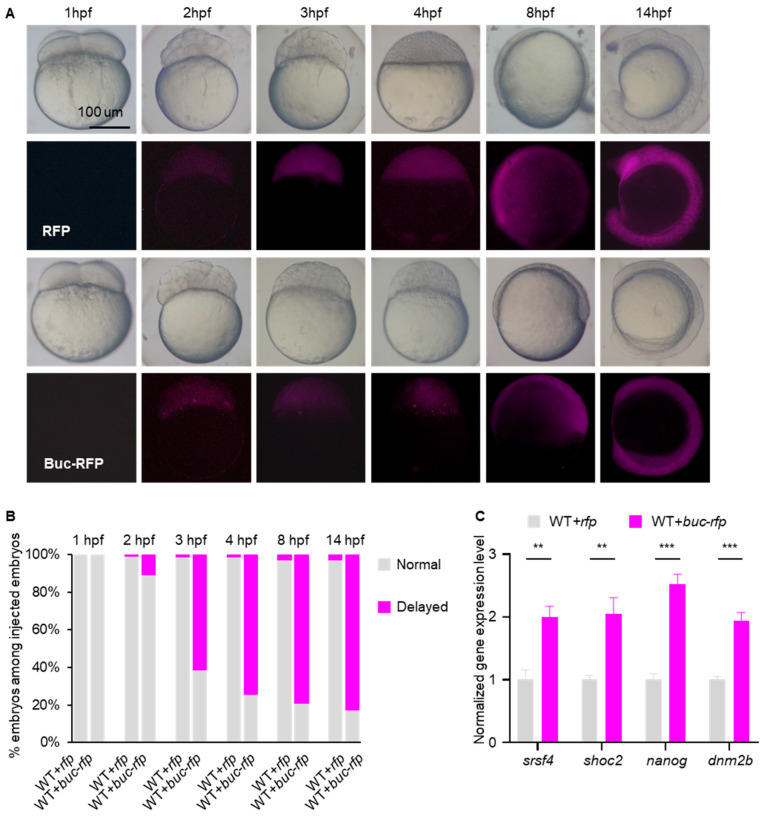
Overexpression of *buc* delays maternal mRNA decay and embryo development. (**A**) Time-matched images revealed that *buc-rfp*-overexpressing embryos displayed a delay in development during early embryogenesis. (**B**) Quantification of phenotypes in *rfp-* and *buc-rfp*-overexpressed embryos during embryogenesis, as indicated in (**A**). (**C**) qRT-PCR analysis of maternal mRNA levels in *rfp-* or *buc-rfp*-overexpressing embryos at 3 hpf. mRNA level was normalized to control values (*rfp*-overexpressing embryo). Error bars represent mean ± S.D.; *n* = 3. *p* values were calculated by two-sided Student’s *t*-test. ** *p* < 0.01 *** *p* < 0.001.

To elucidate the function of Buc during zebrafish embryogenesis, we first examined whether endogenous Buc protein expressed in embryo. Western blot analysis using a Buc-specific antibody detected Buc expression in WT embryos from 1 hpf (hours post fertilization) to 24 hpf, indicating that the protein persists throughout early embryonic development (Figure S1A). In addition, we performed immunofluorescence staining in WT embryos using the Buc antibody and observed Buc expression during early embryogenesis (Figure S1B). Next, we overexpressed *buc-rfp* by injecting *buc-rfp* mRNA into 1-cell WT embryos. No distinct fluorescence signals were detected in *buc-rfp-* or *rfp*-overexpressing embryos at 1 hpf. Between 2 hpf and 4 hpf, embryos overexpressing *buc-rfp* exhibited significant fluorescent granules, whereas control embryos displayed diffuse fluorescence. By 8 hpf, both *buc-rfp-* and *rfp*-overexpressing embryos showed uniform red fluorescence without discernible granule-like structures (Figure 1A). Notably, both *buc-rfp*-overexpressing and control embryos developed normally up to 1 hpf. However, from the side view of a subset of *buc-rfp*-overexpressing embryos, the cell mound appeared noticeably lower at 2 hpf compared to controls, a difference that became more pronounced by 3 hpf. At 4 hpf, the control embryos continued shortening along the animal–vegetal axis, generating a late blastula with a smooth, approximately spherical shape. In contrast, the blastula of *buc-rfp*-overexpressing embryos eventually acquired a smoothly outlined ellipsoidal shape when viewed from the side, and the pre-gastrulation developmental delay persisted until at least 14 hpf (Figure 1A,B). Furthermore, we selected several maternal mRNAs and validated their expression levels by qRT-PCR. These maternal transcripts exhibited significantly higher expression levels in *buc-rfp*-overexpressing embryos compared to control embryos (Figure 1C).

### 3.2. Mbuc Embryos Display Accelerated Decay of Maternal mRNA and Severe Defects in Embryo Development

**Figure 2:**
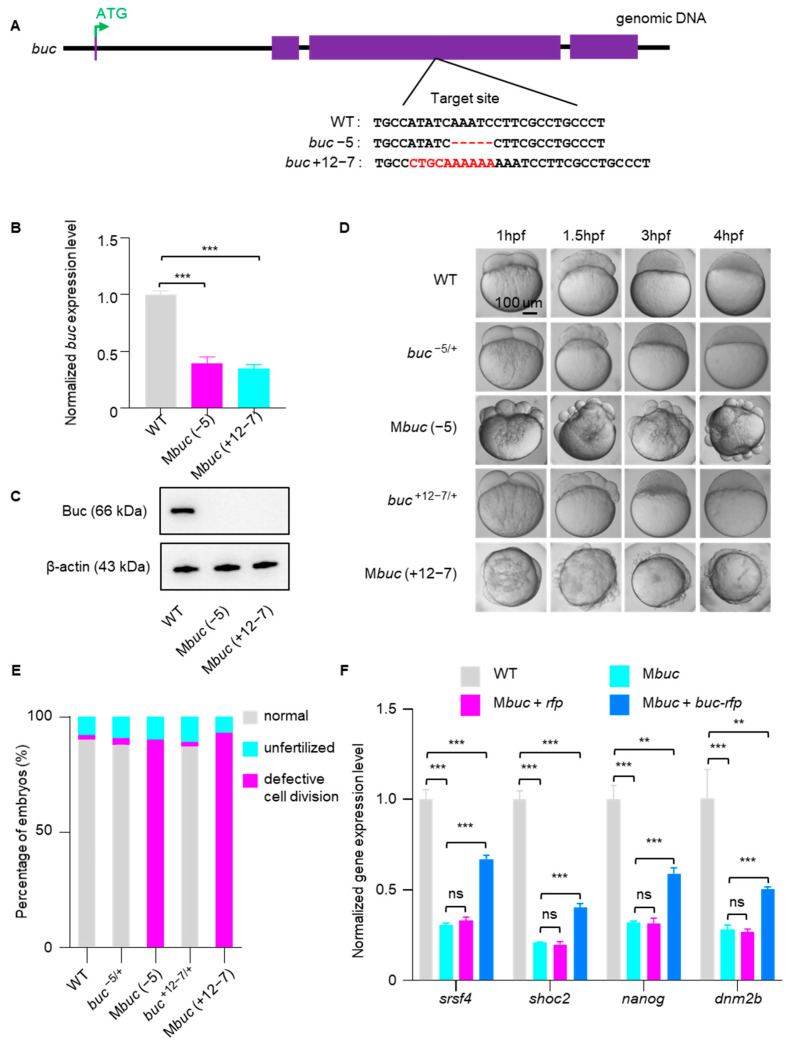
Maternal mRNA undergoes accelerated decay in M*buc* embryos. (**A**) Diagram summarizing the CRISPR/Cas9 editing scheme for *buc* and the types of induced mutations. Purple boxes indicate exons, and the sgRNA target sequence is shown. (**B**) qRT-PCR showing the expression level of *buc* mRNA in WT and M*buc* embryos at 3hpf. (**C**) Western blot revealing Buc protein expression in WT and M*buc* embryos at 3hpf. (**D**) Developmental time course of WT, *buc^−/+^* (crossing heterozygous *buc^−/+^* females with WT males), and M*buc* (crossing homozygous *buc^−/−^* females with WT males) embryos. (**E**) Statistical analysis of developmental phenotypes in WT, *buc^-/+^*, and M*buc* embryos. (**F**) qRT-PCR showing the expression level of maternal RNA in WT, M*buc*, M*buc+rfp*, and M*buc+buc-rfp* embryos at 3hpf. Error bars represent mean ± S.D.; *n* = 3. *p* values were calculated by two-sided Student’s *t*-test. *** *p* < 0.001; ** *p* < 0.01. ns, not significant.

To further investigate Buc’s function in embryos, we used CRISPR/Cas9 to successfully generate *buc* mutant lines by targeting the third exon of the gene (Figure 2A). Through sequencing screening, we identified two mutant alleles in the *buc* locus, one with a 5 bp deletion and another with a 7 bp deletion plus a 12 bp insertion (Figure 2A). qRT-PCR analysis further revealed that *buc* transcript levels were markedly reduced in M*buc* compared with WT embryos, suggesting the occurrence of nonsense-mediated mRNA decay (Figure 2B). Consistent with this, expression analysis by Western blot assay using the Buc antibody showed that Buc protein was undetectable in M*buc* embryos (Figure 2C). Moreover, M*buc* embryos exhibited severe developmental abnormalities during early embryogenesis [22,23], and heterozygous embryos were morphologically comparable to WT embryos (Figure 2D,E). These results collectively demonstrated that we successfully generated a loss-of-function *buc* mutant. Next, we performed qRT-PCR to measure the expression of a set of maternal mRNAs in M*buc* embryos. The results showed that disruption of *buc* function results in a markedly accelerated mRNA decay during early embryogenesis (Figure 2F). In addition, we performed rescue experiments in M*buc* mutant embryos. The results showed that the developmental defects of M*buc* embryos could not be rescued by injection of *buc-rfp* mRNA at the 1-cell stage (Figure S1A,B), but the accelerated degradation of maternal mRNAs was partially restored (Figure 2F). These findings further support the role of Buc in regulating maternal mRNA stability during early embryogenesis.

### 3.3. Buc Overexpression Decelerates the Decay of Bulk Maternal mRNA

**Figure 3:**
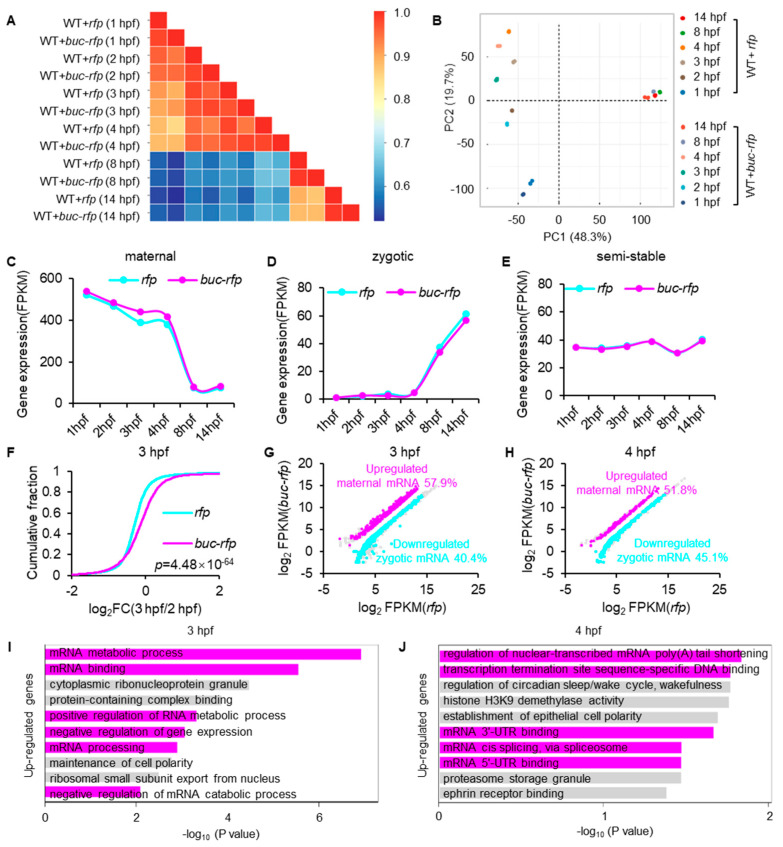
Overexpression of *buc* decelerates the decay of maternal mRNA. (**A**) Heatmap showing the developmental trends of transcriptomes in *rfp-* or *buc-rfp*-overexpressing embryos during early development. (**B**) Principal component analysis (PCA) of RNA-seq samples. (**C**) The abundance of maternal mRNA during early embryo development. The median of maternal mRNA abundance was used for plotting. (**D**) Expression profiles of zygotic genes by their RNA abundance over time as determined by RNA-seq. (**E**) Expression profiles of semi-stable genes by their RNA abundance over time, as determined by RNA-seq. (**F**) Cumulative frequency of maternal mRNA log_2_ fold changes in *buc-rfp*-overexpressing and control embryos from 2 hpf to 3 hpf. The *p* values were calculated using two-sided Wilcoxon and Mann–Whitney tests. (**G**,**H**) Scatter plots showing the enrichment of differentially expressed genes in *buc-rfp*-overexpressing and control embryos at 3 hpf (**G**) and 4 hpf (**H**). The percentages of significantly downregulated zygotic genes (cyan dots) or upregulated maternal genes (magenta dots) are shown. (**I**,**J**) Functional annotation of differentially expressed genes in 3 hpf (**I**) and 4 hpf (**J**). The terms associated with RNA processing are shown in magenta.

To investigate the relationship between Buc and maternal mRNA clearance, RNA-seq was performed for multiple stages of zebrafish embryos. We categorized the transcriptome data into two developmental categories: the MZT period and the post MZT period. Hierarchical clustering (Figure 3A) and principal component analysis (PCA) (Figure 3B) revealed that transcriptomic profiles of embryos injected with either *rfp* or *buc-rfp* underwent marked changes during the MZT, whereas transcriptomic variation was minimal after this transition. Notably, during the MZT, embryos overexpressing *buc-rfp* exhibited pronounced transcriptomic divergence from the control group, a difference that was no longer apparent in the post MZT stage. These findings suggest that *buc-rfp* overexpression specifically influences the transcriptomic dynamics of the MZT but not those of later developmental stages.

To further investigate whether Buc regulates maternal mRNA stability, transcripts were classified into three superclusters (maternal, semi-stable, and zygotic) according to their expression dynamics over time (Figure 3C–E). Importantly, embryos overexpressing *buc-rfp* exhibited both slower maternal mRNA decay and delayed zygotic gene activation (Figure 3C,D), and the expression level of semi-stable genes was consistent with the control (Figure 3E). Differential expression analysis revealed that the most notable change in maternal RNAs occurred around 3 hpf (Figure 3C). From 2 hpf to 3 hpf, maternal mRNAs remained markedly more abundant than in control embryos, suggesting that overexpression of *buc-rfp* led to a deceleration of maternal mRNA clearance (Figure 3F). We found that upon *buc-rfp* overexpression, maternal mRNA accounted for 57.8% and 51.8% of the significantly upregulated genes at 3 hpf and 4 hpf, respectively, while zygotic genes accounted for 40.4% and 45.1% of the significantly downregulated genes, respectively (Figure 3G,H). Moreover, we identified all statistically enriched terms for differentially expressed genes (DEGs) based on 3 hpf and 4 hpf. Genes upregulated in *buc-rfp*-overexpressing embryos were markedly enriched for mRNA metabolic process (Figure 3I,J). Collectively, these findings indicate that Buc overexpression increases the stability of maternal mRNA at 3 hpf.

### 3.4. Buc Enhances the Stability of Igf2bp3-Targeted Maternal mRNAs

**Figure 4:**
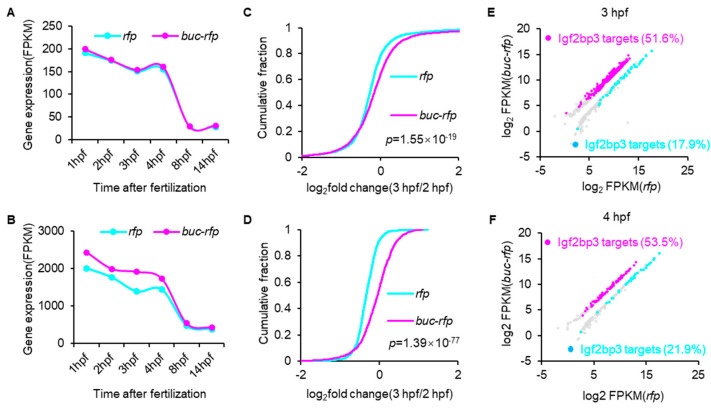
Buc enhances the stability of the target maternal mRNAs of Igf2bp3. (**A**,**B**) Expression profiles of the non-Igf2bp3-target (**A**) or Igf2bp3-target (**B**) maternal mRNAs in *rfp-* or *buc-rfp*-injected embryos from 1 hpf to 14 hpf, respectively. (**C**,**D**) Cumulative frequency of log_2_ fold changes of non-target (**C**) or target (**D**) maternal mRNAs of Igf2bp3 in *rfp-* or *buc-rfp*-injected embryos from 2 hpf to 3 hpf. The *p* values were calculated using two-sided Wilcoxon and Mann–Whitney tests. (**E**,**F**) Scatter plots showing the enrichment of dysregulated maternal genes in *buc-rfp*-overexpressing and control embryos at 3 hpf (**E**) and 4 hpf (**F**). The significantly upregulated (magenta dots) or downregulated (cyan dots) maternal mRNA targets of Igf2bp3 are shown.

To explore the mechanism underlying Buc-mediated maternal mRNA stabilization, we focused on Igf2bp3, which has been extensively implicated in GP assembly and maternal mRNA maintenance [21]. Given Buc’s lack of intrinsic RNA-binding domains, we hypothesized that Igf2bp3 acts as a functional mediator of Buc in protecting maternal mRNA. To investigate whether Buc regulates maternal mRNA degradation through Igf2bp3, we classified maternal mRNAs into two categories: Igf2bp3-targets and non-Igf2bp3-targets. Subsequently, we compared the expression dynamics of these two maternal mRNA groups between *buc-rfp*-overexpressing embryos and the control groups. Compared to non-Igf2bp3-targeted maternal mRNA, degradation of Igf2bp3-targets was noticeably delayed upon *buc-rfp* overexpression (Figure 4A,B). Comparative analysis of mRNA abundance changes between Igf2bp3-targets and non-Igf2bp3-targets from 2 hpf to 3 hpf revealed that Igf2bp3-targeted maternal mRNAs exhibited significantly greater stability compared to non-Igf2bp3-targets in control embryos (Figure 4C,D). Moreover, we found that upon *buc-rfp* overexpression, the percentage of significantly upregulated maternal mRNAs that Igf2bp3 targets was much higher than that for the downregulated counterparts, with 51.4% versus 17.9% at 2 hpf and 53.5% versus 21.9% at 3 hpf (Figure 4E,F). These results suggest that overexpression of *buc-rfp* delays the degradation of maternal mRNAs, most of which are known targets of Igf2bp3.

### 3.5. Buc Keeps Maternal mRNA Stability via Igf2bp3 Regulation

**Figure 5:**
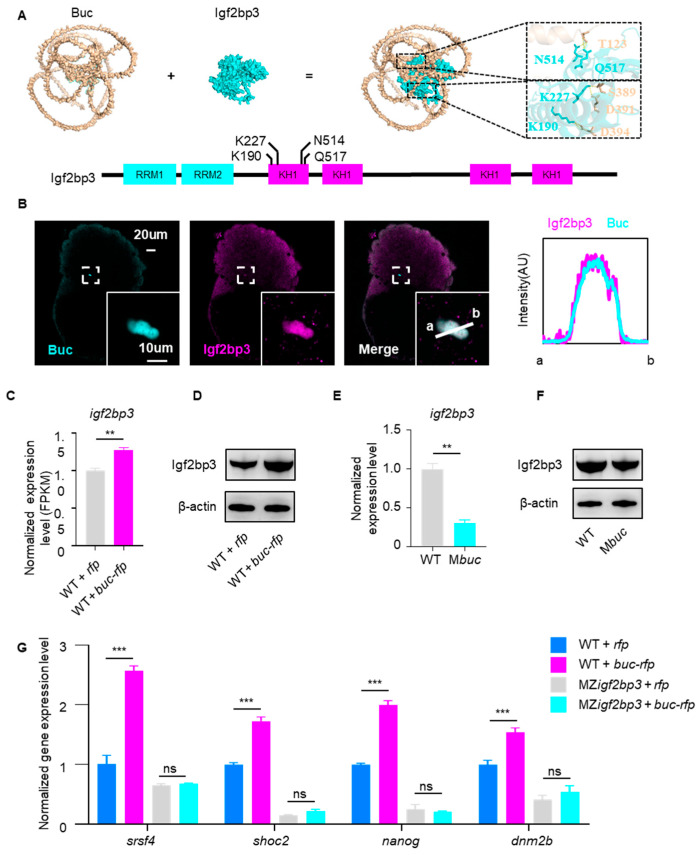
Buc keeps maternal mRNA stability via regulation of Igf2bp3 expression. (**A**) The molecular docking analysis between Igf2bp3 and Buc. The domains (cyan: RRM domain; magenta: KH domain) of Igf2bp3 protein are shown. (**B**) The localization of Buc and Igf2bp3 in WT embryos at 3 hpf. Line graphs display fluorescence intensity for Buc (cyan) and Igf2bp3 (magenta). a-b indicates the line-scan path drawn across the colocalized region for measuring fluorescence intensity along the line. (**C**) *igf2bp3* expression in *buc-rfp*-overexpressing and control embryos at 3 hpf by RNA-seq. Error bars represent mean ± S.D. *p* values were calculated by two-sided Student’s *t*-test, with ** *p* < 0.01. (**D**) WB revealing the expression of Igf2bp3 protein in *buc-rfp*-overexpressing and control embryos at 3 hpf. (**E**) qRT-PCR showing the expression level of *igf2bp3* in WT and M*buc* embryos at 3 hpf. Error bars represent mean ± S.D. *p* values were calculated by two-sided Student’s *t*-test, with ** *p* < 0.01. (**F**) WB revealing the expression of Igf2bp3 protein in WT and M*buc* embryos at 3 hpf. (**G**) qRT-PCR showing the expression level of maternal mRNA level in the embryos at 3 hpf, with WT+*rfp*, WT+*buc-rfp*, MZ*igf2bp3*+*rfp*, or MZ*igf2bp3*+*buc-rfp*, respectively. The mRNA expression level was normalized to values of WT. Error bars represent mean ± S.D.; *n* = 3. *p* values were calculated by two-sided Student’s *t*-test. *** *p* < 0.01. ns, not significant.

Having established Igf2bp3 as a key effector in Buc-mediated mRNA stabilization, we subsequently investigated how Buc regulates the expression of Igf2bp3. The results showed that Igf2bp3 interacts with Buc via its KH domain by molecular docking analysis (Figure 5A). Consistent with this, immunofluorescence imaging at 3 hpf showed clear co-localization of endogenous Buc and Igf2bp3 (Figure 5B). Transcriptomic analyses revealed that *igf2bp3* mRNA levels were significantly increased in *buc-rfp*-overexpressing embryos (Figure 5C). Correspondingly, Western blot results demonstrated an increased protein expression level of Igf2bp3 upon *buc-rfp* overexpression (Figure 5D). Conversely, in *buc* homozygous mutants, *igf2bp3* mRNA and Igf2bp3 protein were markedly downregulated (Figure 5E,F). These results indicate that Buc positively regulates the transcription and translation of *igf2bp3*, thereby ensuring the availability of Igf2bp3 for maternal mRNA stabilization.

To further investigate the role of Igf2bp3 in Buc-mediated maternal mRNA stability, we overexpressed *buc-rfp* in maternal–zygotic *igf2bp3* (MZ*igf2bp3*) (crossing homozygous *igf2bp3^−/−^* females with *igf2bp3^−/−^* males) mutants and quantified the maternal mRNA expression levels via qRT-PCR. The results showed that *buc-rfp* overexpression failed to maintain maternal mRNA stability in MZ*igf2bp3* embryos, in contrast to its effect in WT embryos (Figure 5G). Collectively, these findings demonstrate that Buc maintains maternal mRNA stability and early embryogenesis through an Igf2bp3-dependent mechanism during MZT.

## 4. Discussion

The MZT represents a critical developmental phase in vertebrate embryogenesis, characterized by the systematic degradation of maternal mRNA and the concomitant initiation of zygotic genome transcription [2,3,4,5,6,7]. A tightly regulated mechanism governing the stability of maternally deposited mRNA is essential for proper progression of early embryonic development. GP factors have been identified to maintain mRNA stability, thereby underlying PGC fate determination [10,11,12,24,25]. However, whether GP factors such as Buc function in regulating maternal mRNA stability for embryo development remains unclear. In this study, we elucidate the molecular mechanisms of the GP organizer Buc in regulating maternal mRNA stability during zebrafish embryogenesis, revealing its functional dependency on the RNA-binding protein Igf2bp3. Our findings provide new insights into the mechanisms controlling mRNA stability during early embryogenesis.

Our results reveal that overexpression of the GP organizer Buc markedly delayed maternal mRNA degradation and embryonic development in zebrafish (Figure 1). By contrast, M*buc* embryos exhibited severe development defects and accelerated degradation of maternal mRNA (Figure 2). This phenotype reflects the developmental consequences of delayed maternal mRNA clearance, similar to those observed when mRNA degradation pathways are disrupted. In zebrafish, loss of the m^6^A “reader” Ythdf2 function decelerated decay of m^6^A-modified maternal mRNA and caused cell cycle arrest and developmental delay [26]. Likewise, disruption of the m^5^C reader Ybx1 compromised maternal mRNA stability and early embryogenesis [27], and depletion of *igf2bp3* destabilized maternal mRNA and led to severe developmental defects [20]. The regulation of maternal mRNA on embryogenesis may be a conserved pathway across species. For instance, loss of maternal Argonaute-2 (Ago2) function hindered maternal mRNA degradation, which results in embryonic development arrest at the 2-cell stage in mouse [28,29]. In Drosophila, Smaug regulates maternal mRNA clearance, and deletion of *smaug* leads to a slowed cell cycle in early embryos [30,31,32]. In Drosophila, mutations in *osk* (which encodes the GP organizer) result in loss of polarity and are ultimately lethal [33,34]. Similarly, loss of CSR-1 function impaired hundreds of maternal mRNA decay instances and led to embryonic lethality in *C. elegans* [35,36,37]. Together, these studies suggest that maternal mRNAs are essential for early embryonic development and that dysregulation of maternal mRNA degradation may trigger various defects, including cell cycle disorder, loss of polarity, and embryonic lethality.

Our data showed that Buc’s function in mRNA regulation was mainly concentrated in the period of MZT during early embryo development (Figure 3A,B), with the greatest differences in maternal mRNA levels observed at 3 hpf (Figure 3C). Consistent with previous studies [11,23,38,39], we found that Buc formed obvious granules in early embryo. In addition, our data showed that the appearance of Buc granules coincides with the timing of the MZT (Figure 1A), suggesting that Buc granules may contribute to the MZT. Buc contained intrinsically disordered and prion-like domains (analogous to Xenopus Xvelo1) that promoted phase separation and the formation of ribonucleoparticles (RNPs) [40,41]. This RNP condensate physically sequesters specific mRNAs and their associated RBPs away from the bulk cytoplasm. In early zebrafish embryos, Buc was transported to the cleavage furrows by the kinesin motor Kif5Ba and posited there to be nucleate GP [42]. In effect, Buc-mediated compartmentalization bundles maternal mRNA (such as *nanos*, *vasa*, *dnd1*) into granules [40]. Because many of these mRNAs would ordinarily be targeted for degradation at the MZT (e.g., via the massive *miR-430* program), concentrating them in a Buc-controlled GP compartment shields them from degradation [43,44]. In summary, Buc-dependent GP RNPs create a microenvironment that safeguards germline maternal mRNAs during the MZT, thereby coupling germ cell fate to maternal mRNA regulation.

In the process of Buc-mediated maternal mRNA stabilization, the RNA-binding protein Igf2bp3 serves as a key mediator (Figure 4). Furthermore, Igf2bp3 interacts and co-localizes with Buc to form granules in early embryos (Figure 5). We proposed that Buc formed the architectural scaffold of the GP granule, while Igf2bp3 served as the RNA-binding engine that selected and preserved the maternal mRNA within that scaffold. This coordinated action underlies the stable inheritance of GP content and proper specification of PGCs in zebrafish, mirroring previous studies in GP regulation [21]. In species with maternal inheritance mode, a common mechanism to protect mRNAs from degradation is to incorporate them into maternally provided RNP granules [11,25,43,45,46], although the specific regulatory factors involved may differ between species. In Drosophila, the PIWI protein Aubergine (Aub) was shown to bind directly to the germline-specific poly(A) polymerase Wispy, promoting polyadenylation and stabilization of mRNAs within the GP [47], whereas in *C. elegans*, CGH-1 forms complexes with translational regulators and selected maternal mRNAs, thereby preventing their degradation [48,49]. In this study, we demonstrated that Buc maintains maternal mRNA stability by upregulating the expression of Igf2bp3, which may be in a unique m^6^A-dependent pathway in vertebrates [21]. Overall, our results support a model in which germ plasm factors (Buc and Igf2bp3) play a central role in maintaining maternal mRNA stability during vertebrate embryogenesis.

It should be noted that the primary conclusions of this study are derived from the *buc* overexpression model, which provides direct evidence that Buc regulates maternal mRNA stability through Igf2bp3-mediated mechanisms. The *buc* mutant model was used only as a complementary loss-of-function approach to support this finding. Although overexpression of *buc* during early embryogenesis delays maternal mRNA degradation and consequently slows embryo development, analyzing *buc* loss-of-function phenotypes remains challenging due to defects that originate during oogenesis. Specifically, *buc* mutants exhibit oocyte-stage abnormalities such as excess micropyles and disrupted polarity [22,23], which can lead to polyspermy and loss of embryonic polarity. As a result, the phenotypes observed in early embryos may partly reflect secondary consequences of oogenic defects. At present, it is difficult to selectively disrupt *buc* function only during embryogenesis while completely avoiding the secondary influence of oocyte abnormalities. Nevertheless, the complementary results obtained from both *buc* overexpression and loss-of-function models consistently support Buc’s role in maintaining maternal mRNA stability, providing robust evidence for the proposed regulatory mechanism.

## 5. Conclusions

Our study demonstrates that the GP organizer Buc orchestrates the MZT in zebrafish, in part by upregulating Igf2bp3 to stabilize maternal mRNAs, thereby supporting proper early embryogenesis. Overexpression of *buc* delays maternal mRNA clearance and causes a developmental delay, whereas *buc* knockout accelerates mRNA degradation and leads to severe defects in early zebrafish embryos. Moreover, Buc co-localizes with Igf2bp3 and enhances its expression in early embryos, thereby protecting key maternal mRNAs from degradation at 3 hpf. These findings expand Buc’s functional repertoire and highlight its pivotal role in preserving maternal mRNA stability for normal embryonic development.

## Figures and Tables

**Table 1:** Primer sequences for qRT-PCR.

Gene Name	Primer Sequence (5′-3′)	Purpose
*srsf*-F	CCGAGATGGTGGCAACAG	qRT-PCR
*srsf*-R	CCTGTAATCTGTGCGTGTCG	qRT-PCR
*shoc*-F	TCCATCTGTTGCCCTCGTC	qRT-PCR
*shoc*-R	TGGTGATGCGGTTGAAGC	qRT-PCR
*nanog*-F	GGCGTCCCGAATCTGAG	qRT-PCR
*nanog*-R	CCGTTCTGCGAGTGTCCC	qRT-PCR
*dnm2b*-F	TTCCCTCCAGACCCACT	qRT-PCR
*dnm2b*-R	TCGGACGGATGATTGTG	qRT-PCR
*igf2bp3*-F	AGCGAGTGGAGGGATTTCA	qRT-PCR
*igf2bp3*-R	ATTGACGCACCAGCGAAGC	qRT-PCR
*buc*-F	CCACAAGTGACCCAAGAGCG	qRT-PCR
*buc*-R	CCTACCACCACCAACATAAACA	qRT-PCR
*β-actin*-F	CGAGCAGGAGATGGGAACC	qRT-PCR
*β-actin*-R	CAACGGAAACGCTCATTGC	qRT-PCR

## Data Availability

Further information and requests for any data reported in the manuscript should be directed to the corresponding author J.M. (jmei@ihb.ac.cn). Code: This study did not generate any original code. This study did not generate new unique reagents. Further information and requests for resources such as reagents listed in the key resources table should be directed to the corresponding author J.M. (jmei@ihb.ac.cn).

## Supplementary Materials

The following supporting information can be downloaded at: https://www.mdpi.com/article/10.3390/cells14231879/s1, Figure S1. Expression and localization of endogenous Buc during zebrafish embryogenesis. Figure S2. Rescue analysis of M*buc* embryos at 3 hpf. Table S1. Minimal dataset.
