# Supplementary material for: Buc Maintains Maternal RNA Stability and Embryogenesis in Zebrafish
Source: Cells. 2025 Nov 27;14(23):1879. doi: 10.3390/cells14231879 (PMC12691124; doi:10.3390/cells14231879)
Supplement: Supplementary file 1 [file cells-14-01879-s001.zip › supplementary figures.pdf]

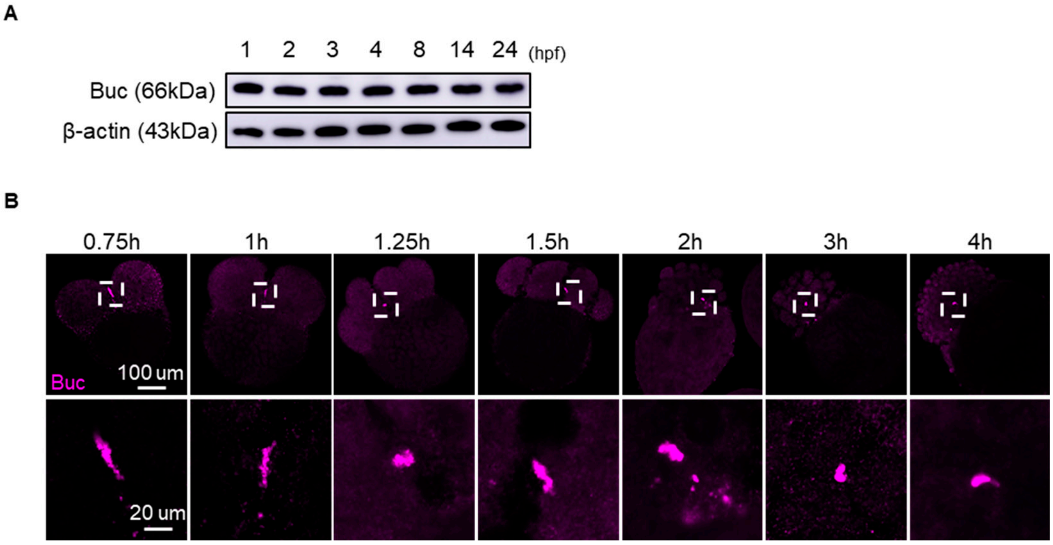

Figure S1. Expression and localization of endogenous Buc during zebrafish embryogenesis. (A)Western blot revealing Buc protein expression in WT embryos at various developmental stages. (B) Time-matched fluorescence images showing that endogenous Buc protein in zebrafish embryos.

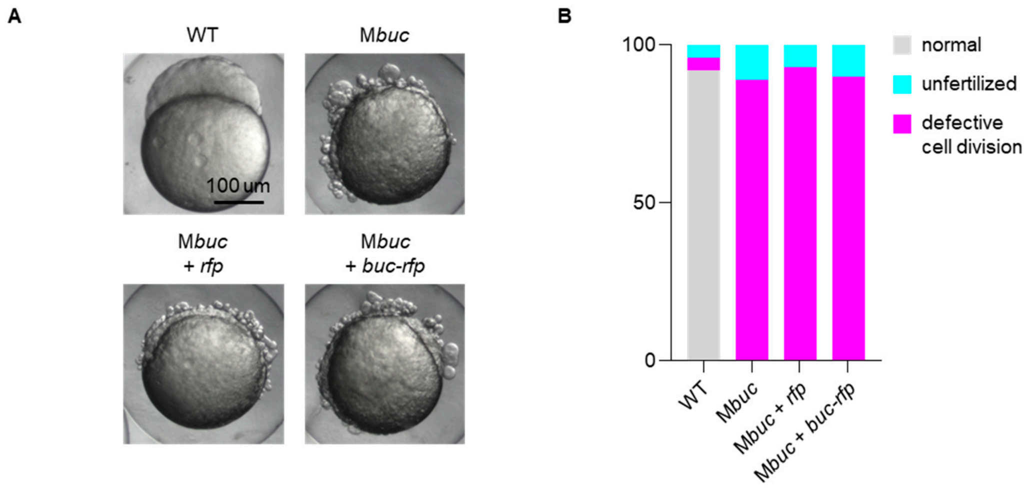

Figure S2. Rescue analysis of Mbuc embryos at 3 hpf. (A) The image of WT, M*buc*, M*buc*+*rfp* and M*buc*+*buc-rfp* embryos at 3hpf. (B) Statistical analysis of developmental phenotypes in WT, M*buc*, M*buc*+*rfp* and M*buc*+*buc-rfp* embryos at 3hpf.
